# Exploring factors influencing maternal willingness to donate breast milk: a descriptive qualitative study

**DOI:** 10.3389/fgwh.2026.1785890

**Published:** 2026-06-03

**Authors:** Yanxi Liu, Xue Xia, Xiaoqian Zeng, Yao Lu, Xiaoli Yan, Min Liang

**Affiliations:** The First Affiliated Hosptial of Army Medical University, Chongqing, China

**Keywords:** breast milk, breast milk bank, postpartum woman, qualitative research, women

## Abstract

**Objective:**

Breast milk donation is a critical intervention to improve neonatal nutritional security, especially for high-risk infants such as preterm and low-birth-weight babies, yet China's breast milk donation system is underdeveloped with a stark gap between maternal donation willingness and actual behavior. The aim of this study was to explore the factors influencing maternal breast milk donation intentions in China.

**Methods:**

In a tertiary hospital in Chongqing, China, 15 women expressed their facilitators and in hibitors of breast milk donation. The results of the interviews were analyzed individually through Nvivo software in a phenomenological approach, emphasizing the depth of each participant's experience.

**Results:**

Four main themes emerged regarding the factors influencing maternal willingness to donate breast milk. Theme 1, “Maternal own cognitive factors”; Theme 2, “Social support and environmental factors”; Theme 3, “Information access and dissemination factors”; and Theme 4: “Donation process factors”.

**Conclusion:**

The factors affecting Chinese mothers’ willingness to donate breast milk involve not only personal perceptions, but also the embedded influence of domestic social and cultural contexts, with the donation process emerging as a particularly critical factor. Therefore, it is recommended that healthcare professionals target the aforementioned four dimensions, streamline the breast milk donation process and improve maternal awareness of breast milk donation, thereby boosting the breast milk donation rate in China.

## 1 Introduction

Breastfeeding, as the gold nutritional standard for infant growth and development, not only provides ideal nutritional ratios and immune protection for newborns, but is also recognized as a key measure to reduce the incidence of necrotizing enterocolitis (NEC) and the risk of infection in newborns (1). The World Health Organization (WHO) clearly recommends donor breastmilk as the preferred form of replacement feeding when mothers are unable to perform hand-feeding due to illness, separation of mother and baby, or insufficient lactation (2). The establishment of the Human Milk Bank (HMB) system has provided an important way to address the plight of the 78 million babies worldwide who are unable to be breastfed each year (3). By standardizing the collection, screening, storage and distribution of donor breastmilk, the Breastmilk Bank provides early-life nutritional security, especially for high-risk groups such as preterm and low-birth-weight infants, and significantly reduces their complication and mortality rates (4).

However, despite the maturity of international breastmilk banks, the development of China's breastmilk donation system still faces serious challenges. Studies have shown (5) that although 74%–76.7% of the mothers in Nanning Maternal and Child Hospital expressed their willingness to donate, the actual donation rate was less than 3%. This huge gap between willingness and behavior exposes the structural deficiencies of the current breast milk donation support system in China, and urgently requires an in-depth exploration of the deeper motivations affecting maternal decision-making. There are fewer domestic studies on donation intentions, mostly focusing on quantitative analyses that reveal the extent of maternal knowledge about breast milk donation. Hu Shuhong et al (6) showed that only 27.4% of the respondents knew about breast milk banks or donation channels, and the degree of knowledge was significantly positively correlated with the place of residence, literacy level and family income. This finding was further validated in the group of mothers of infants up to 6 months of age: the knowledge rate in this group was 34.1%, but the actual donation rate was only 2.6%, and factors such as age, number of births, and history of preterm birth significantly influenced their knowledge level (7). The above studies show that maternal knowledge of breastmilk donation is currently low in China, and that the influencing factors investigated through cross-sectional surveys only relate to their general demographic and obstetric factors, and have not yet been explored in depth to determine the role of values, external support, traditional culture, and other factors in influencing them. In addition, the search revealed that there are fewer qualitative empirical studies on Chinese mothers’ willingness to donate breastmilk, and only one Hong Kong scholar explored breastfeeding mothers’ perceptions of breastmilk donation and the establishment of a breastmilk bank in Hong Kong in 2015 (8). However, due to the special characteristics of the Hong Kong region, there may be some differences between the life backgrounds and perceptions of mothers in this region and those of women in the mainland region, and thus the results may not provide a complete picture of the level of knowledge that influences breastmilk donation among Chinese mothers.

International research has also carried out some studies on breast milk donation. A phenomenological study in the Limpopo province of South Africa (9) showed that maternal acceptance of donor breast milk was influenced by both HIV stigma and traditional taboos: Respondents were significantly hesitant to use donor breast milk due to fear of transmission of the virus, while cultural beliefs such as ancestor worship gave breast milk sharing a supernatural risk significance. In contrast, research in Europe and the United States has highlighted the psychological motivation for altruism and trauma repair. For example, a large-scale survey in the United Kingdom (2) found that successful donors had a positive psychological experience (pride, sense of worth) through the act of breastfeeding, which particularly helped to alleviate negative emotions resulting from childbirth trauma, neonatal intensive care (NICU) experiences, or infant mortality, an emotional healing effect that suggests a potential impact of the act of donation on maternal mental health.

Moreover, domestic qualitative research on obstetric nurses has also shed light on the barriers to breast milk donation in China. A qualitative study on nurses’ barriers to breast milk bank operation indicated that family members’ traditional taboos regarding “milk leakage” among lactating women may act as a key psychological barrier to breast milk donation (10). However, this study only explored nurses’ perceptions and observations, and lacked direct first-hand evidence from lactating mothers themselves, making it impossible to accurately reflect the real thoughts and decision-making mechanisms of mothers regarding breast milk donation.

Therefore, this study aims to explore the following research question: What are the key factors influencing the willingness of mainland Chinese mothers to donate breast milk, and what are the underlying cognitive and cultural mechanisms? To answer this question, the study utilized an interpretive phenomenological research design, aiming to gain in-depth insight into the complex experiences and core influencing factors of Chinese mothers’ willingness to donate breast milk.

## 2 Method

### 2.1 Participants

This study adopted purposive sampling, a core qualitative sampling method, which was appropriate as it targeted postpartum women with potential insights into breast milk donation (meeting the inclusion criteria) to deeply explore subjective experiences and influencing factors—consistent with the interpretive phenomenological research aim of capturing context-specific perspectives. This study was conducted from June to August 2025 in a purposive sample of obstetric wards in a tertiary care hospital in Chongqing, China. Inclusion criteria: (1) 18 years of age or older; (2) Born and raised primarily in mainland China; (3) Fluent in Chinese and willing to share personal experiences with the interviewer; (4) Postpartum women with no prior experience of breast milk donation (the study focused on exploring the donation intention of mothers who had not engaged in breast milk donation behavior, to accurately capture the original perceptions and potential influencing factors of the target population). Exclusion criteria: (1) Women who were seeking treatment for mental disorders; (2) Women with heart disease, hypertension, thalassemia, or severe pregnancy complications (these conditions may affect maternal lactation function, breast milk quality, and physical recovery status, which are core factors related to breast milk donation willingness; meanwhile, severe physical diseases may lead to biased subjective cognition and emotional expression of the research topic); (3) Women who were unwilling to share personal experiences.

### 2.2 Develop the interview guide

Based on the research objectives, a preliminary interview outline was drafted through literature review and group discussions. Prior to formal interviews, two pre-interviews were conducted with patients. Based on the findings, the formal interview outline was revised and finalized as follows.

### 2.3 Procedure

This study was approved by the Medical Ethics Committee of the tertiary care hospital where the research was conducted (approval number: BIIT2025262KX), and written permission was obtained from the hospital's obstetric department and nursing management team for the implementation of the study, including the use of interview venues and the recruitment of participants in the obstetric ward. Reflexivity considerations were addressed during the research process. The lead interviewer was a female master's student with training in obstetric and reproductive health research, who underwent standardized training on interview techniques, emotional regulation, and ethical communication. A neutral, non-threatening relationship was established with participants to reduce social desirability bias.

Participants were identified and recruited with the assistance of the head nurses and clinical nurses of the obstetric ward. First, nurses screened hospitalized postpartum women who met the inclusion criteria based on clinical medical records and daily nursing assessments; then, the research team introduced the study purpose, procedures, and ethical norms to the eligible women face-to-face, and invited voluntary participation. The nursing staff were trained by the research team on recruitment criteria and communication norms in advance to ensure the accuracy of subject screening and avoid improper guidance. All recruitment and research processes were conducted in compliance with the Health Insurance Portability and Accountability Act (HIPAA) regulations: all medical record information used for screening was de-identified, and the research team only obtained basic demographic information with the written informed consent of the participants, with no unauthorized access to private medical data. Women who agreed to participate were asked to fill out a demographic questionnaire and were then interviewed in a special conversation room. During the interview, participants were informed of their right to refuse any question and to terminate the interview at any time. The interviews were conducted face-to-face using a bottom-up approach.

To ensure the validity of the interview methodology, a series of pilot interviews were conducted with 2 women, which are not reported in this study. After each pilot interview, questions were revised and refined to ensure comprehensiveness. For the sake of convenience and to minimize the additional time burden on the participants, the interviews were arranged to coincide with their visits to the hospital for routine postnatal rehabilitation check-ups. During the interview, the interviewer maintained an objective approach and avoided making any personal assumptions or judgments about the women's pregnancy experiences. The researcher reflected on potential influences such as educational background and gender, ensuring that interpretations remained grounded in participants’ own narratives rather than the researcher's preconceptions. The interviews lasted between 20 and 30 min. The final version of the interview outline is shown in Table 1.

**Table 1:** Interview guide.

Serial number	Problem
1	Have you heard of “breast milk donation”? In what context did you hear about it?
2	Where do you primarily learn about breast milk donation?
3	Do you understand the specific process for donating breast milk?
4	Do you know if there is a human milk bank in this area? Or do you know who ultimately receives donated breast milk?
5	What is your primary feeling when you think about “donating your own breast milk”?
6	How do you feel about your breast milk being used to nourish another baby? What emotions does this evoke in you?
7	What factors do you believe would encourage or motivate you to donate breast milk?
8	When deciding whether to make a donation, what factors do you primarily consider?

### 2.4 Data analysis

Data saturation was confirmed as no new codes or themes emerged after the 12th interview, validating the adequacy of the 15-participant sample. The audiotapes of the interviews were transcribed within two weeks of the interviews. Data were analyzed using a phenomenological approach based on Giorgi's (11) methodology as follows. First, the interviews were transcribed verbatim over a two-week period and read repeatedly to familiarize themselves with the content. Line-by-line coding using NVivoPlus® was used by the first author to summarize themes related to breast milk donation. These codes were then categorized into “meaning units” that represented key aspects of the participants’ experiences. The meaning units were further distilled into overarching themes and sub-themes to capture common and unique perspectives. To ensure the validity of the findings, emerging themes were reviewed and discussed by the research team, and any discrepancies were resolved through consensus.

The interview transcripts were reviewed repeatedly in order to gain insight into the factors influencing breastmilk donation among postpartum women. All statements related to breast milk donation were extracted and translated from Chinese to English by the lead interviewer. A rigorous translation and back-translation process was utilized to ensure the accuracy and authenticity of the translations. First, a bilingual person reviewed the initial translation to ensure that the meaning was accurately conveyed and adapted to spoken English. Subsequently, a second independent translator, unfamiliar with the original Mandarin, translated the English version back into Mandarin. Finally, the research team compared the original transcripts, translations, and back-translations to address any discrepancies and to ensure that the final English translations accurately captured the nuances of the participants’ experiences.

## 3 Results

Fifteen participants were selected through purposive sampling for interviews. The interviewees ranged in age from 27 to 37 years old, with approximately half having delivered via cesarean section. Gravida and Para (GxPy) and mode of delivery were included as key demographic variables because pregnancy history and delivery mode are closely associated with maternal lactation function, physical recovery status and postpartum psychological state—all of which are core factors influencing breast milk donation willingness and capacity. Detailed demographic characteristics of the participants are presented in Table 2.

**Table 2:** Characteristics of semi-depth interview participants.

Serial number	Age	Gravida & Para (GxPy)	Educational attainment	Occupation	Mode of delivery
1	28	G_1_P_1_	Associate degree	Freelance	Natural childbirth
2	29	G_3_P_1_	Undergraduate	Freelance	Natural childbirth
3	37	G_4_P_1_	Undergraduate	Freelance	Cesarean section
4	37	G_4_P_1_	Undergraduate	Teacher	Cesarean section
5	32	G_1_P_1_	Undergraduate	Teacher	Cesarean section
6	31	G_1_P_1_	Undergraduate	Accounting	Cesarean section
7	33	G_1_P_1_	Undergraduate	Military personnel	Natural childbirth
8	30	G_1_P_1_	Master's degree	Staff member	Cesarean section
9	27	G_1_P_1_	Undergraduate	Staff member	Natural childbirth
10	36	G_1_P_1_	Associate degree	Unemployed	Cesarean section
11	31	G_2_P_1_	Undergraduate	Staff member	Cesarean section
12	32	G_1_P_1_	Master's degree	Healthcare workers	Cesarean section
13	36	G_2_P_1_	Undergraduate	Staff member	Cesarean section
14	33	G_2_P_2_	Undergraduate	Healthcare workers	Cesarean section
15	29	G_3_P_1_	Undergraduate	Healthcare workers	Cesarean section

GxPy: x = Gravida (number of pregnancies), y = Para (number of live births); Freelance: Engaged in non-fixed occupational work with flexible working models; Staff member: Full-time administrative or professional staff in enterprises and public institutions.

### 3.1 Theme 1: maternal self-awareness

#### 3.1.1 Maternal knowledge and understanding of breast milk donation

Maternal knowledge and understanding of breast milk donation affected their willingness to donate. The results of the interviews revealed that most of the mothers had a low level of knowledge about breastmilk donation, with their cognition limited to the basic understanding that donated breastmilk would be used to help other newborns with insufficient breastmilk supply. Few mothers had knowledge about the specific links of the donation process, such as the collection, storage, transportation, and testing of breastmilk. This superficial cognitive level was reflected in the vague expressions of most participants, for example, N10 (Unemployed, Cesarean section) stated: “*I'm not sure about this, I haven't learned about it”*, and N11 (Staff member, Cesarean section) expressed a relatively one-sided understanding: “*I feel like it should just be for little babies that are kind of hospitalized, maybe just born, and then the mother doesn't have quite enough milk, and then some donations for them.”*

However, despite the low level of knowledge about breast milk donation, most of the mothers indicated that they were willing to donate their excess breast milk, and they uniformly regarded this act as a continuation of motherhood. This emotional perception transcended their limited cognitive level, as N4 (Teacher, Cesarean section) said: “*37 Degrees of Love, every mom has a really deep love for her kids. Whether it's another mom's breastmilk for my child or my breastmilk for another child, it's really a wonderful expectation for the next generation and a great desire to give them better.”* N5 (Teacher, Cesarean section) also emphasized the sense of responsibility behind the act: “*If I can solve the problem of feeding small children, I think it's a responsibility a love, an extension and continuation of motherhood.”* For the specific group of premature infants, mothers showed a stronger willingness to help, with N3 (Freelance, Cesarean section) mentioning: “*For those premature babies who need breastmilk but their mothers can't provide breastmilk this situation is very relevant and I think it's caring as a mother.”*

#### 3.1.2 Self-body sensations and perceptions

Mothers’ assessment of their physical recovery and their subjective perception of the quantity and quality of breastmilk they produced affected their willingness to donate. Participants who held a positive evaluation of their physical state and lactation capacity showed a higher willingness to donate, and this assessment was the direct basis for their donation intention. Among the participants with positive physical assessment, their willingness to donate was accompanied by a strong sense of pride in sharing, for example, N9 (Staff member, Natural childbirth) said: *“Honestly from the bottom of my heart, if my milk hadn't been affected, I definitely would have brought it in for donation right away.”* N11 (Staff member, Cesarean section) also linked physical recovery to the willingness to share: “*Because I recovered pretty well after giving birth, I had quite a lot of milk, and all I felt that being able to share my extra milk with other babies who didn't have enough was something that made me feel proud.”*

In contrast, mothers who had negative perceptions of their physical state or lactation capacity showed obvious hesitation in donation willingness, and their concerns mainly focused on insufficient milk supply and questionable milk quality. N1 (Freelance, Natural childbirth) expressed anxiety about milk quantity: “*Definitely there was a little bit of hesitation because I would be worried that my own baby would not have enough to eat and then I would have to donate again, and I was quite worried that the amount would not be enough. At the same time, because my own baby just seems to be used to my way of eating, people in Chongqing like to eat spicy food, and then I'm also drinking coffee.”* N8 (Staff member, Cesarean section) was concerned about the potential impact of their physical state on milk quality and the health of recipient babies: “*Because it's hard with babies, just not enough sleep. Still worrying about adverse reactions to my breastmilk for other children, and donating it would be worrying about other babies in case their bodies don't adapt, etc.”*

#### 3.1.3 Psychological factors

Psychological aspects such as maternal compassion, altruism, and concern about whether the act of donating would adversely affect them and their babies influenced their willingness to donate. Mothers with a strong sense of compassion and altruism took the initiative to associate their excess breastmilk with the needs of other babies, and this psychological drive directly enhanced their donation willingness. N10 (Unemployed, Cesarean section) combined her professional knowledge with altruistic feelings: “*Because of my profession, I think I have some knowledge about breastfeeding. Then I feel that breastfeeding a child will always make me feel better, and I always feel that breastmilk is more nutritious than formula milk. If I have more, I would like to share this feeling with others.”* N6 (Accounting, Cesarean section) paid attention to the actual feeding difficulties of specific babies: “*Sometimes there are some babies he doesn't like formula, so having this channel to help them makes me feel pretty good.”* N2 (Freelance, Natural childbirth) expressed a direct altruistic intention: “*I think it's great to be able to help others. I would definitely donate if I had any extra myself.”*

However, some mothers had psychological concerns about the negative impact of donation, which became the main psychological barrier to their donation willingness, and their concerns were all centered on the safety of their own babies and their own physical comfort. N11 (Staff member, Cesarean section) prioritized the nutritional needs of her own baby: “*The main concern is that babies don't get enough to eat, and your own child's nutrition definitely comes first.”* N1 (Freelance, Natural childbirth) was worried about the physical discomfort caused by the donation process: “*Because breastmilk would have been sucked out of the breast pump as well, and sucking too long and too much makes your nipples sore.”*

### 3.2 Social support and environmental factors

#### 3.2.1 Family support

Mothers indicated that the attitudes of family members (e.g., husbands, parents, etc.) toward maternal breast milk donation and the support they gave affected their willingness to donate breast milk. The intergenerational differences in family attitudes were particularly obvious, and the conservative views of the elders became the main family barrier to donation for many participants. N7 (Military personnel, Natural childbirth) mentioned the opposition from the elderly in the family: “*The elders in the family are very traditional and definitely worry that their grandchildren won't have enough to eat, so that makes it difficult for me.”* N11 (Staff member, Cesarean section) also stated the practical considerations of family members: “*The family might think you have so much milk, surely you should keep it for yourself ah, not only can you drink it for a long time, but you can also save a large amount of money.”* In contrast, participants with open family values received positive family support, which became a strong backing for their donation willingness, as N5 (Teacher, Cesarean section) said: “*My family should support me without affecting the feeding of my child because our family is still very open-minded.”*

#### 3.2.2 Socio-cultural context

The socio-cultural background influenced the willingness of mothers to donate, and the most obvious influencing factors were the level of education and occupational background, which directly shaped the mothers’ perceptions of breast milk donation. Participants with professional backgrounds in maternal and child health or high educational attainment expressed more positive and open perceptions, and their professional knowledge helped them overcome traditional cognitions about breast milk. N8 (Staff member, Cesarean section) linked education and occupational exposure to donation willingness: “*I think it has to do with your education and the people you are exposed to around you. I'm a nurse and I know relatively more about this, so I might just be more willing to donate.”*

In contrast, participants with non-medical backgrounds or lower educational attainment were bound by traditional beliefs, and they regarded breast milk as a private product or had concerns about the potential risks of donation, which impeded their donation willingness. N6 (Accounting, Cesarean section) considered breast milk donation a private topic: “*I might only donate to someone when they make that request, because it's a very private topic after all, and maybe if you donate to a stranger on your own accord, people will worry about whether you're doing it for some bad purpose instead.”* N7 (Military personnel, Natural childbirth) expressed concerns about the quality and safety of donated breast milk: “*Some people may be concerned about the quality of donated breast milk and whether it will affect the health of other people's babies.”* N10 (Unemployed, Cesarean section) also mentioned the risk aversion mindset of ordinary citizens without medical knowledge: “*If I were an ordinary citizen and didn't have this layer of knowledge underpinning me as a medical practitioner, I might feel like taking mine to someone else, so in case someone else eats it and there's something wrong with it, that it will not come to haunt me in that kind of mindset.”*

#### 3.2.3 Medical environment and nursing support

The promotion and guidance of breastmilk donation in the hospital or healthcare facility where the mother gave birth, as well as the information and guidance provided by healthcare staff, affected her willingness to donate. Proactive guidance and operational support from healthcare staff significantly enhanced mothers’ donation willingness, as the professional explanation of medical staff effectively reduced mothers’ doubts about donation. N11 (Staff member, Cesarean section) mentioned the positive role of nurse guidance in the breastfeeding clinic: “*Just like the breastfeeding clinic nurses might talk about how if they say they have extra milk, they can make a donation to help other babies, and after hearing what they have to say we might be more willing to donate.”* N9 (Staff member, Natural childbirth) was introduced to breast milk donation through nurses and thus generated donation willingness: “I would have been freshly dissected and had no milk. The nurse said I could use donated breast milk if I needed it. Then I was introduced to breastmilk donation and I thought I would like to do that if I could to help more babies.” N10 (Unemployed, Cesarean section) also emphasized the sense of reassurance brought by professional explanation: “*After the medical staff have explained this to me, I will definitely be more comfortable with this when I understand it in more detail.”*

On the contrary, the lack of relevant promotion and guidance in the medical environment made mothers unable to obtain effective information about donation, thus missing the opportunity to generate donation willingness. N6 (Accounting, Cesarean section) described the lack of medical guidance during her postpartum hospitalization: “*Because I had a cesarean section at the time, I was rushed out of the hospital after three days of postpartum hospitalization, and no one went to talk to us about it, so there wasn't too much going on to find out about it or pay attention to it.”*

### 3.3 Information access and dissemination factors

#### 3.3.1 Access to information

Different sources of information about breastmilk donation, such as the Internet, hospital promotional materials, verbal information from health care workers, and sharing by other mothers, affected their willingness to donate. Participants had obvious differences in trust in different information sources, and the Internet, as a non-authoritative source, was often questioned for its authenticity, while hospital-based authoritative information sources were more recognized by mothers. N11 (Staff member, Cesarean section) expressed skepticism about Internet information: “I've read on the internet that some of those premature babies, or other moms who don't have enough breastmilk, receive breastmilk donations, or moms who have a lot of milk donate breastmilk, but I'm not sure if I'm going to believe what I've read on the internet.” In contrast, N7 (Military personnel, Natural childbirth) showed trust in hospital publicity: “Early on there was a talk about breastfeeding in the hospital about breastmilk donation, and I felt that since it was organized by the hospital it was safe and would make me feel comfortable about doing this.”

#### 3.3.2 Accuracy and adequacy of information

Whether the information a mother received about breastmilk donation was accurate and complete affected her willingness to donate. Inaccurate or incomplete information would lead to mothers’ doubts about the safety of donation, while accurate and sufficient information could reduce their worries and enhance their donation willingness. Most participants’ doubts focused on the technical links of donation, such as donor screening, milk storage and testing, and these doubts were all due to the lack of accurate information. N9 (Staff member, Natural childbirth) expressed skepticism about the safety of the donation process due to insufficient information: “*I was skeptical about breastmilk donation, and I saw a video on a public website in our hospital that talked about breastmilk donation. I was thinking, first of all, the mother's physical health, and then the safety of his milk, such as temperature control, bacteria, all kinds of can meet the standard, this is the most direct. And then another thing is how well the hospital keeps the breastmilk, actually I still have a big question mark.”* N6 (Accounting, Cesarean section) was worried about the safety of the post-donation process: “*My concern is safety. While I can guarantee that my donated breastmilk is safe, I don't know a lot about the process after I donate it out, so I'm afraid that if my own breastmilk is given to someone else's baby, it will be hard to tell if something goes wrong with him.”* N11 (Staff member, Cesarean section) also focused on the screening of infectious diseases: “*It's just trying to get on top of the screening of the breast milk, that kind of infectious disease might be a concern.”*

Only participants who believed that hospitals could provide accurate and standardized information had full trust in the donation process, as N10 (Unemployed, Cesarean section) said: “*I think hospitals organizing this should routinely go for screening for at least those diseases. Diseases that are contagious this kind of or those that can be transmitted through breast milk, surely you can't have that, and those safety can definitely be guaranteed.”*

### 3.4 Donation process factors

#### 3.4.1 Complexity of the donation process

The complexity of the donation process and the tediousness of the collection process affected maternal willingness to donate, and most participants expressed concerns about the operational complexity of donation, which they thought would interfere with their daily postpartum life. N11 (Staff member, Cesarean section) mentioned the general concern about the cumbersomeness of collection: “*Maybe it's just a concern that the collection aspect will be cumbersome and interfere with your life.”* N10 (Unemployed, Cesarean section) was worried about whether the home storage and collection methods met the donation criteria: “*If I were to donate, I'd be worried about whether my storage would meet the criteria for donation, because I just have a regular refrigerator at home, and I'm not sure if I'm storing it the right way, or the way I'm collecting it.”* N9 (Staff member, Natural childbirth) was unclear about the specific donation process and expressed hesitation about the potential complexity: “*I don't know if this donation process is one where they come to the house or suck it out at home and bring it to the hospital? This process would be very distracting to my willingness to donate if it's too complicated.”*

#### 3.4.2 Cost factors associated with donations

Mothers incurred certain costs in the process of breastmilk donation, such as the time consumed in the donation process and the transportation costs to deliver breastmilk to the hospital, and the level of these costs and the ability of the mother to bear them affected her willingness to donate. Postpartum mothers were mainly responsible for taking care of their babies, so time cost was their primary concern, and they considered the time spent on donation as a heavy burden. N1 (Freelance, Natural childbirth) linked time cost to baby care: “*I would be concerned about the ease of the donation process because I need to take care of my child at home right now, so it wouldn't be very convenient in terms of time if I needed to take my breastmilk to the hospital.”* N8 (Staff member, Cesarean section) also mentioned the dual burden of time, energy and economic costs: “*I think the point of time and energy is very important because it's also inconvenient to travel back and forth, and there are all kinds of expenses incurred, is this kind of something that we need to bear?”*

## 4 Discussion

This study explored the multidimensional factors influencing mothers’ willingness to donate breastmilk through qualitative interviews, and four core themes were extracted: mothers’ own perceptions, social support and environmental factors, information acquisition and dissemination factors, and donation process factors. The study found that despite limited knowledge of the specific process and safety of breastmilk donation, many mothers showed a high willingness to donate, largely influenced by emotional drivers (e.g., continuation of maternal love, altruism). However, factors such as insufficient awareness, lack of family support, single access to information, and complex donation processes may hinder their actual donation behavior, revealing cultural and social specificities in breast milk donation behavior.

### 4.1 Multidimensional effects of maternal cognitive systems

This study identified a notable “knowledge-emotion-behavior” paradox in maternal decision-making for breast milk donation: most mothers (e.g., N10, N6) expressed strong donation willingness despite limited knowledge of specific donation procedures. This paradox is clearly explained by the combined application of the Health Belief Model (HBM) and Theory of Planned Behavior (TPB) (12, 13), two classic health behavior frameworks, which respectively reveal the psychological and behavioral logic of mothers’ donation intentions.

From the HBM perspective, mothers’ donation decisions depend on a trade-off between their perceived benefits of donation, perceived barriers, and self-efficacy in their own lactation capacity—three core elements of the model. Our findings show that Chinese mothers perceived significant benefits of breast milk donation: they recognized it could nourish vulnerable infants such as preterm babies, and the emotional value of “extending maternal love” (N4, N5) further strengthened their willingness, which became the key driver of their donation intention even with poor procedural knowledge. This emotional drive is highly consistent with the traditional Chinese moral of “caring for one's own children as well as those of others” and forms a localized donation motivation model (14). Meanwhile, mothers’ main perceived barriers focused on safety risks and personal costs: N9's doubts about breast milk storage and testing standards reflected process safety concerns, while N1's worry about nipple soreness from pumping and N11's fear of insufficient milk for their own baby reflected physical and child-rearing burdens. Such cognitive deficits over donation safety easily lead to an “intention-behavior gap”, a result that is particularly prominent in the Chinese context where widespread societal concerns over food safety amplify maternal risk perceptions (15, 16). In addition, mothers’ self-efficacy was closely tied to their body perception: those who believed they had recovered well and produced sufficient, high-quality milk (e.g., N11) had higher self-efficacy and stronger willingness, while those with negative evaluations of their lactation (e.g., N1, N8) had lower self-efficacy and showed obvious hesitation. Notably, Chinese mothers exhibit a unique “dual assessment” in body perception: they biomedicalize breastmilk (i.e., regard it as a nutritional substance meeting biomedical standards) as described in Western studies, but are also heavily influenced by the concept of “constitution” in traditional Chinese medicine (17) (e.g., N1's worry that spicy food and coffee intake would affect milk quality), and this superimposed effect of Eastern and Western perceptions forms a distinctive perspective for breast milk donation research in China.

From the TPB perspective, donation intention is shaped by three interrelated factors: attitude toward the behavior, subjective norms, and perceived behavioral control—all of which were reflected in our study. Mothers held a positive attitude toward donation: driven by altruism and traditional Chinese morals (e.g., N2, N6), they viewed donation as a meaningful act. Subjective norms had little impact on their intention, as most mothers had little contact with breast milk donation in daily life and thus lacked external social or group guidance on donation, making their intention driven more by internal emotions than external pressure. The most prominent factor was perceived behavioral control, whose deficits highlight the unique parenting pressures faced by Chinese mothers under the nuclear family structure (18): many mothers had strong willingness but low perceived control due to practical constraints, such as N11's concern about cumbersome donation procedures, N1's worry about time costs of hospital delivery, and N10's doubt about whether home storage met standards. These doubts made mothers feel they lacked the ability and resources to complete donation smoothly, directly hindering the transformation of intention into actual behavior. Moreover, the altruistic motives of Chinese mothers (N2, N10) not only contain universal pro-social tendencies but also reflect the special sense of intergenerational responsibility formed against the backdrop of China's fertility policy (18).

These unique cognitive characteristics rooted in Chinese social and cultural contexts indicate that breast milk donation promotion strategies in China cannot simply copy international experience, but must be formulated based on an in-depth understanding of local cultural psychology and fertility norms.

### 4.2 Structural role of social support systems

The mechanism of structural influence of social support systems on maternal breast milk donation intentions presents a distinctive Chinese socio-cultural feature. In the dimension of family support, it was found that maternal donation decision-making in China was deeply influenced by traditional family concepts, resulting in a unique pattern of “intergenerational decision-making”: as shown in cases N7 and N11, the conservative perception of breastmilk as the “nutritional capital of the family” by elders contrasted sharply with the spousal-dominated decision-making pattern of the Western nuclear family. This difference stems from the Chinese family's unique “differential order pattern”, the distribution of decision-making power along the intergenerational axis (18, 19). It is worth noting that the modern model of family support demonstrated by the N5 case reflects the transformation of family values in contemporary China, where maternal acceptance of donations stems not only from the knowledge base, but is also related to the notion of body image they receive (20). In terms of medical support, the reliance of Chinese mothers on professional guidance (N9, N11) is both consistent with the universal laws of behavioral change theory (21) and specific to the institutional context: in the current situation of uneven distribution of healthcare resources, the difference in the level of support provided by tertiary hospitals vs. primary healthcare facilities makes it challenging to localize the application of the HMBANA criteria in China (22, 23). These studies suggest that breastmilk donation promotion strategies in China need to build a “family-society-healthcare” support network that takes into account urban-rural differences and intergenerational attitudinal changes, as well as the reality of a hierarchical treatment system.

### 4.3 Information ecosystem optimization needs

This study analyzes the critical influence of the information ecosystem on maternal breastmilk donation decision-making, and reveals the information dissemination patterns and cognitive mechanisms with Chinese characteristics. In the dimension of information channels, it was found that Chinese women who gave birth in China showed unique “system-dependent” information acquisition characteristics (24): for example, N7's trust in hospital publicity and N11's skepticism about Internet information not only confirmed the general rule of “authoritative source effect”, but also reflected the “trust preference for public institutions” formed under the Chinese healthcare system (25). The deeper reason for this tendency, in contrast to the more decentralized model of information access in Western societies, lies in the institutional trust that has developed over time in our healthcare system. It is worth noting that the multichannel communication strategy advocated by WHO faces special challenges in the Chinese context: on the one hand, it has to adapt to the digital ecosystem dominated by WeChat and other “mega-apps”, and on the other hand, it has to take into account the disparity in information accessibility brought about by the urban-rural digital divide (26).

At the level of risk perception, the questioning of the safety of N6 and N9 goes beyond the classical risk perception framework proposed by Slovic (27), and presents the “double anxiety” unique to the transition period of Chinese society: it contains both uncertainty about the technical process (e.g., testing standards), and implicitly worries about the implementation of the system. This composite risk perception is closely related to the collective memory formed by the frequent food safety incidents in China in recent years (28).

Therefore, this study suggests that China's information intervention strategy needs to build a dual-track “authority-interaction” model: strengthening the leading role of traditional authoritative sources, such as medical institutions, and developing participatory communication adapted to the new media ecosystem, as well as designing differentiated risk communication programs for different education and age levels.

### 4.4 Practical optimization of the donation process

This study thoroughly explores the key role of donation process optimization in enhancing maternal breastmilk donation intentions, revealing practical dilemmas and improvement paths with Chinese characteristics.

In the convenience dimension, the time cost concerns reflected in cases such as N1 and N8 highlight the more complex convenience challenges facing maternity in China's urbanization: On the one hand, the long commuting time in big cities aggravates the time pressure; on the other hand, the burden of multi-child care under the two-child policy further increases the behavioral cost, and this “double time cost” phenomenon is a unique obstacle to the promotion of breastmilk donation in China (29).

In terms of standardization, N10's questioning of storage standards not only reflects the lack of technical specifications, but also exposes the regional imbalance in the quality of medical services in China. Although the EMBA standard provides an international reference, direct application faces localization challenges: the differences in cold chain logistics brought about by the vastness of China, the gap in testing capacity between regions and other practical factors need to be taken into account (30).

Therefore, the results of this study suggest that process optimization in China should adopt a “gradient advancement” strategy: in developed cities, we can pilot the “smart donation” model (integrating community cold-chain sites and mobile medical platforms), and in less-developed areas, we should prioritize the establishment of a basic standardized system (referring to the Code of Practice for Breastmilk Banking 2022), and at the same time, we should combine the ethics of mutual help in traditional culture with the development of a community support network (10, 31).

### 4.5 Limitations and strengths

This study has certain limitations: the purposive sampling of 15 participants from a single tertiary hospital in Chongqing led to geographic and institutional bias, and the sample with a high proportion of highly educated mothers and healthcare workers limits the generalizability of the findings to the broader maternal population in China. In addition, face-to-face interviews may have caused social desirability bias, and the cross-sectional design only explored breast milk donation intentions rather than actual behaviors, failing to track the intention-behavior transformation process. Notwithstanding these limitations, the study has important strengths: as a rare qualitative empirical study on breast milk donation willingness among mainland Chinese mothers, it fills the domestic research gap by using an interpretive phenomenological approach to deeply explore maternal subjective experiences and cognitive mechanisms, with rigorous data analysis ensuring the credibility of the results. Moreover, the study identifies four culturally specific core influencing factors and integrates the Health Belief Model and Theory of Planned Behavior to explain the unique “knowledge-emotion-behavior” paradox in Chinese mothers’ decision-making, revealing localized characteristics of breast milk donation in China. These findings provide targeted empirical and theoretical references for optimizing the domestic breast milk bank system and formulating donation promotion strategies, and also lay a theoretical foundation for subsequent quantitative research on this topic.

## 5 Conclusions

This descriptive qualitative study elucidated the multifaceted factors that influence maternal willingness to donate breast milk in China. The findings suggest that maternal willingness to donate is not a monolithic concept, but is dynamically shaped by the interplay of intrinsic and extrinsic factors. Maternal self-awareness was an underlying theme in which limited knowledge of donation protocols coexisted with strong altruistic motives (e.g., “37 Degrees of Love”) stemming from maternal identity. Physiological self-perceptions-such as confidence in breast milk adequacy/quality and recovery status-directly moderated willingness to donate, while psychological concerns about infant nutrition or personal discomfort posed significant barriers.

Social support systems had a crucial impact on decision-making: family attitudes (supportive or oppositional), sociocultural norms (level of education, professional exposure, and traditional beliefs about breast-milk intimacy), and institutional support (guidance from health-care providers) were critical, and a lack of proactive health-care advocacy significantly reduced opportunities for participation. In addition, the accessibility and credibility of information played a decisive role: credible information from health-care providers increased confidence, while ambiguous or inaccessible details cast doubt on safety and the rigor of the procedure. Crucially, pragmatic constraints—including perceived complexity of donation logistics, time burdens, and ancillary costs—often outweighed altruistic inclinations. Women prioritized their infant's well-being and personal capacity, indicating that willingness alone is insufficient without structural enablers.

These findings necessitate multilevel interventions: (1) Implementing educational initiatives to clarify donation procedures, safety protocols, and benefits during prenatal/postnatal periods; (2) Integrating donor support into healthcare systems through nurse-led counseling and logistical aid; deploying family engagement strategies to address socio-cultural taboos; (3) Simplifying donation logistics (e.g., home collection). Ultimately, cultivating a supportive donation environment requires aligning maternal altruism with structural enablers to ensure informed, accessible, and sustainable participation.

## Data Availability

The raw data supporting the conclusions of this article will be made available by the authors, without undue reservation.
